# Cognitive profiles in bipolar I disorder and associated risk factors: Using Wechsler adult intelligence scale—IV

**DOI:** 10.3389/fpsyg.2022.951043

**Published:** 2022-10-06

**Authors:** Hayoung Ko, DongYeon Park, Jaehyun Shin, Rina Yu, Vin Ryu, Wonhye Lee

**Affiliations:** ^1^Department of Psychology, Virginia Polytechnic Institute and State University, Blacksburg, VA, United States; ^2^Department of Psychiatry, National Center for Mental Health, Seoul, South Korea; ^3^School of Education, Virginia Polytechnic Institute and State University, Blacksburg, VA, United States; ^4^Department of Mental Health Research, National Center for Mental Health, Seoul, South Korea; ^5^Department of Clinical Psychology, National Center for Mental Health, Seoul, South Korea

**Keywords:** Wechsler Intelligence Scale-IV (WAIS-IV), bipolar I disorder, cognitive performance, risk factors, redundancy analysis

## Abstract

**Background:**

Despite the growing evidence of cognitive impairments in bipolar disorder (BD), little work has evaluated cognitive performances utilizing the latest version of the Wechsler Intelligence Scale-IV (WAIS-IV), which is one of the most widely used neurocognitive assessments in clinical settings. Furthermore, clinical characteristics or demographic features that negatively affect the cognitive functioning of BD were not systematically compared or evaluated. Accordingly, the present study aimed to examine the cognitive profile of bipolar I disorder (BD-I) patients and associated risk factors.

**Methods:**

Participants included 45 patients, diagnosed with BD-I, current or most recent episode manic, and matching 46 healthy controls (HC). Cognitive performance was evaluated *via* WAIS-IV, and clinical characteristics of the BD-I group were examined *via* multiple self- and clinician-report questionnaires.

**Results:**

Multivariate analysis of covariance (MANCOVA) results indicated that the BD-I group demonstrated significantly poorer performance compared to the HC group in subtests and indexes that reflect working memory and processing speed abilities. Redundancy analysis revealed that overall symptom severity, manic symptom severity, and anxiety were significant predictors of cognitive performance in BD-I, while age of onset, past mood disorder history, depression severity, and impulsiveness showed comparatively smaller predictive values.

**Conclusion:**

The current study suggests cognitive deterioration in the cognitive proficiency area while generalized ability, including verbal comprehension and most of the perceptual reasoning skills, remain intact in BD-I. The identified risk factors of cognitive performance provide specific clinical recommendations for intervention and clinical decision-making.

## Introduction

Bipolar spectrum disorders (BD) are one of the major chronic mental disorders with an aggregate lifetime prevalence of 2.4%, including bipolar I disorder (BD-I), bipolar II disorder (BD-II), and subthreshold BD (Merikangas et al., 2011). In recent epidemiologic studies, BD has been consistently found to be a severe chronic disorder with frequent relapses (Geddes and Miklowitz, 2013; Ferrari et al., 2016). It is also associated with high suicidality rates, high unemployment rates, and low productivity (Zimmerman et al., 2010; Undurraga et al., 2011; Malhi et al., 2013; Pompili et al., 2013; Razzouk, 2017; Hakulinen et al., 2019). Considering that the treatment cost per person for BD is the highest among the general medical and psychiatric diseases, the socioeconomic burden is also considered to be high (Revicki et al., 2005; Kleine-Budde et al., 2014; Cloutier et al., 2018; Bessonova et al., 2020). In particular, cognitive impairment has been increasingly recognized as a common factor in BD (Torrent et al., 2012; Kozicky et al., 2013), and damage has been found in attention, working and episodic memories, processing speed, and executive function domains, which are shown to be significantly lower than healthy controls (HC; Cardoso et al., 2015; Cotrena et al., 2016). These cognitive deficits are a major factor in the deterioration of quality of life and psychosocial adjustments, such as job performance, interpersonal relationships, and life satisfaction (Godard et al., 2011; Duarte et al., 2016; Gitlin and Miklowitz, 2017). Furthermore, the impairment is known to persist even between mood episodes and is associated with a worse prognosis (Malhi et al., 2007; Frías et al., 2017).

Previous studies have shown specific neurocognitive areas of impairment, and researchers have used various cognitive and neurocognitive batteries for their studies (Hsiao et al., 2009). Although previous research provides effect sizes of impairments derived from each cognitive test, different scores from different neurocognitive tests prevents comparison among studies and confirmation of the presence and severity of cognitive impairment (Cullen et al., 2016; Dickinson et al., 2017). Therefore, utilizing a more standardized and widely used cognitive assessment in order to identify the impaired cognitive domains in patients with BD is crucial. The Wechsler Adult Intelligence Scale (WAIS) is a standardized objective cognitive measurement that is widely used around the world as a tool that provides information on the general cognitive function of people with various psychopathologies (Harrison et al., 1988; Camara et al., 2000; Georgas et al., 2003). The recently revised version of WAIS-IV (Wechsler, 2008) is based on theories of intelligence that were current to the WAIS-IV development. It is known that the psychometric properties of the WAIS-IV were improved by further scientific understanding of neurological cognitive functions (McGrew, 2009; Lichtenberger and Kaufman, 2013). However, there is a lack of research on the cognitive profiles or performances of patients with BD measured by the WAIS-IV.

Furthermore, although there is accumulated evidence of cognitive impairments in a substantial number of patients with BD, key risk factors accounting for the specific cognitive deterioration remain unclear (Cullen et al., 2016). For instance, several clinical features have been identified to demonstrate a negative relationship with cognitive function, such as familial history, coexisting medical comorbidities, and clinical features, including the age of onset, presence of psychotic symptoms, severity of illness, and the number of mood episodes (Robinson and Nicol Ferrier, 2006; Tsai et al., 2007; Goodwin et al., 2008; Latalova et al., 2011; Roux et al., 2019); however, thus far, which clinical features or demographic characteristics impact cognitive dysfunction the most in patients with BD has not been systematically evaluated.

### Present study

The present study seeks to investigate the cognitive profiles of patients with BD-I, current or most recent episode manic, and to compare the function between the BD-I and HC groups by utilizing the WAIS-IV to identify each cognitive area. Although previous research has evaluated neurocognitive deterioration in the BD group, there are few studies that have investigated the cognitive profile using the WAIS-IV (i.e., K-WAIS-IV), which is a widely used assessment tool that provides information on four areas of cognitive function: verbal comprehension, perceptual reasoning, working memory, and processing speed. The current study aimed to directly compare the cognitive profile between BD-I and a matching HC group. Specifically, this study only included BD-I patients whose most recent episode or current episode was manic, considering the known heterogeneity of BD group in terms of clinical and cognitive performances (Sweeney et al., 2000; Cardoso et al., 2015). Furthermore, we investigated how multiple predictors that include patients’ mental health history and current clinical characteristics could explain the cognitive performances or deterioration of the BD-I group. Although previous research has identified clinical characteristics that significantly affect the deterioration of neurocognitive function of BD patients, there is still a lack of studies that have accumulated all clinical characteristics and compared which factor impacts specific areas of function.

We hypothesized that (1) BD-I group would show a significant deterioration in working memory and processing speed ability regardless of demographic factors, while demonstrating intact verbal comprehension and perceptual reasoning ability compared to the HC group, and (2) clinical characteristics of both past history and current status would explain the specific cognitive function of BD-I group. In other words, we hope to uncover particular clinical characteristics from the heterogeneous predictor sets that are highly associated with specific cognitive areas from criterion sets, *via* redundancy analyses (RDA).

## Materials and methods

The current study was approved by the Institutional Review Board of the National Center for Mental Health (NCMH; IRB 116271–2018-04) and written informed consent was obtained from all participants prior to enrollment.

### Participants and procedure

This study included two different groups, including 45 individuals diagnosed with bipolar I disorder (BD-I), current or most recent episode manic, and 46 healthy controls (HC) with no lifetime history of any psychiatric disorders. Participants of the BD-I group were recruited from the mood disorder clinic at the NCMH, and they were either in-patient or out-patient clients who contacted the clinic for intervention and/or comprehensive psychological assessment. The diagnostic evaluation was performed using the Mini International Neuropsychiatric Interview (MINI Plus), a semi-structured interview tool based on the diagnostic criteria of the Diagnostic and Statistical Manual of Mental Disorders (DSM-IV; Sheehan, 1998). Diagnostic criteria for BD-I, current or most recent episode manic, from the DSM-5 (American Psychiatric Association, 2013) and previous medical records were additionally used to further confirm the diagnosis. Individuals with any previous or current history of a substance use disorder, neurodevelopmental disorders (e.g., Attention Deficit/Hyperactivity Disorder, Autism Spectrum Disorder, or intellectual disabilities), neurocognitive disorders (e.g., Alzheimer’s disease), or traumatic brain injury were excluded from the analysis. The BD-I group completed both self- and clinician-reported measures and neurocognitive assessment. The participants of the HC group were recruited as a control sample *via* advertisements, flyers, and local community support. As for the BD-I group, diagnostic interviews were administered to all potential participants for the HC group in order to carefully exclude individuals with any history of psychiatric disorders. A total of 46 participants completed a neurocognitive assessment.

### Measures

#### Self- and clinician-report measures

**Clinical Global Impressions Scale-Bipolar Version** (CGI-BP; Spearing et al., 1997). The severity of adaptive dysfunction was assessed using the Clinical Global Impressions Scale-Bipolar version (CGI-BP). The CGI-BP measures dysfunction severity due to depression, mania, and overall BD illness. Severity scores of depression and mania were used for analysis. Scores on the CGI-BP range from 1 to 7, with higher scores indicating greater severity. All three scales are observer rating scales which are designed to be assessed on the basis of the attitudes, behaviors, and answers of the patients.

**Quick Inventory of Depressive Symptomatology-Self Report** (QIDS-SR; Rush et al., 2000, 2003). The QIDS was developed to evaluate depressive symptoms (i.e., sleep, appetite, mood state, vitality, suicidal ideation, concentration) of the past 7 days. The QIDS consists of 16 items rated on a 4-point Likert scale ranging from 0 (symptom not present) up to 3 (most severe symptom). According to the criteria recommended by previous research, higher scores indicate severe depression (i.e., 0–5: no depression, 6–10: mild depression, 11–15: moderate depression, 15–20: severe depression, 21–27: very severe depression). In Korea, the QIDS-SR was validated with Korean patients which showed acceptable psychometric properties in assessing depressive symptoms (Hong et al., 2013).

**Beck Anxiety Inventory** (BAI; Beck et al., 1988). The BAI is a self-report assessment tool developed by Beck et al. to evaluate the occurrence and severity of anxiety symptoms of the past 7 days, and is widely used in both clinical and research settings. Originally, it was created in order to differentiate between depression and anxiety. The BAI consists of 21 items rated on a 4-point Likert scale ranging from 0 (not at all) to 3 (severely). According to the interpretation guidelines, the higher scores indicate more severe anxiety (i.e., 0–7: minimal anxiety, 8–15: mild anxiety, 16–25: moderate anxiety, and 26–63: severe anxiety). In this study, the K-BAI which was published in Korean was used (Lee et al., 2016).

**Mood Disorder Questionnaire** (MDQ; Hirschfeld et al., 2000). The MDQ is a self-report scale developed to evaluate and screen a lifetime history of bipolar disorder including hypomanic and manic symptoms. The MDQ consists of three parts: the first section includes 13 yes/no questions regarding previous hypomanic or manic episodes. The second section has one yes/no question that asks whether the symptoms occurred simultaneously during the same period of time. Finally, the last section consists of one question regarding the influence and interference level due to the symptoms. The author suggested a cutoff score of 7, which indicates 7 or more symptoms occurred at the same time with moderate impairment at the least. A validated version of the Korean MDQ reported an internal consistency of 0.88 and intraclass correlation coefficient of 0.77 (Jon et al., 2009).

**Beck Hopelessness Scale** (BHS; Beck and Steer, 1988). The BHS is a self-report questionnaire developed to assess attitudes toward the perceived future, specifically hopelessness, with 20 items of true/false questions. The 20 items contain both negative and positive statements and the respondent is asked to evaluate their attitude based on the previous week. The total score is the sum of each item and a higher score indicates a severe level of hopelessness. Previous research indicates that hopelessness evaluated via BHS was a predictor of significant suicide intentions, and a cut-off of 9 is suggested (Niméus et al., 1997; Brown et al., 2000). A Korean version of the BHS demonstrates an internal consistency coefficient of 0.85 and test–retest reliability of *r* = 0.86 for an average of 7.2 days (Kim et al., 2015).

**Barratt Impulsiveness Scale-11-Revised** (BIS-11-R; Patton et al., 1995). The BIS is a self-report instrument developed to assess personality traits of and behavioral impulsiveness including planning, self-regulation, impulsive behavior, and attention in everyday life. It consists of 30 items rated on a 4-point Likert scale ranging from 1 (rarely/never) to 4 (almost always/always), and is widely used in both research and clinical settings. The total score and three subscale scores (i.e., attentional impulsiveness, motor impulsiveness, and non-planning impulsiveness) are provided, and research has supported its validity, reliability, and predictive value (Stanford et al., 2009). The Korean version of the BIS-11-R has shown support for its validity and reliability, and was adopted in this study (Lee et al., 2012).

**Biological Rhythms Interview of Assessment in Neuropsychiatry** (BRIAN; Giglio et al., 2009). The BRIAN was used to assess biological physical rhythms such as sleep, physical activity, social activity, and eating patterns over the last 15 days, considering the impact and etiological role of biological rhythm disturbances in bipolar disorder (Takaesu, 2018). This self-report measure consists of 21 items that are evaluated on a 4-point scale from 1 (not at all) to 4 (often). Higher scores indicate greater disturbance in the biological rhythms (Cho et al., 2018).

#### Cognitive assessments

**Korean Wechsler Adult Intelligence Scale-IV** (K-WAIS-IV; Wechsler, 2008; Hwang et al., 2012). Cognitive function was evaluated *via* K-WAIS-IV, a validated Korean version of the WAIS-IV, with 7 indices, 10 core subtests, and 7 process scores. The 7 indices scores included the Full-Scale Intellectual Quotient (FSIQ) that indicates the level of the individual’s overall cognitive function, General Ability Index (GAI) which is comprised of a Verbal Comprehension Index (VCI) and Perceptual Reasoning Index (PRI) scores, and Cognitive Proficiency Index (CPI) based on Working Memory Index (WMI) and Processing Speed Index (PSI) scores. There are four partial sub-indexes, including VCI, PRI, WMI, and PSI, and ten subtests; Similarities (SI), Vocabulary (VC), Information (IN), Block Design (BD), Matrix Reasoning (MR), Visual Puzzles (VP), Digit Span (DS), Arithmetic (AR), Symbol Search (SS), and Coding (CD). Finally, process scores were also included in the present study, which includes Block Design No Time Bonus (BDN), Digit Span Forward (DSF), Digit Span Backward (DSB), Digit Span Sequencing (DSS), Longest Digit Span Forward (LDSF), Longest Digit Span Backward (LDSB), and Longest Digit Span Sequencing (LDSS).

### Statistical analyses

The frequency and descriptive analyses were conducted in the demographic analysis. Significant sociodemographic variables between BD and HC samples were compared using independent *t*-test or chi-squared test, as appropriate. In order to evaluate group differences of WAIS-IV performance scores between the BD and HC groups, multivariate analysis of covariance (MANCOVA) was conducted after controlling the sociodemographic variables, which showed significant differences between the two groups. The partial η^2^ (denoted by η^2^_p_ hereafter) value was presented to measure the effect size. Additionally, a post-hoc Bonferroni-corrected alpha level (*p* < 0.001) was applied to minimize the risk of a type I error when conducting multiple statistical tests.

To evaluate the clinical risk factors associated with cognitive impairment, redundancy analyses (RDA; van den Wollenberg, 1977) were conducted to examine the relationship between criterion (i.e., cognitive functions evaluated *via* WAIS-IV) and predictor (i.e., demographic and clinical characteristics) variables. Specifically, we divided the criterion variable into 3 sets to apply redundancy analysis. The first set includes all 7 index scores (i.e., FSIQ, GAI, CPI, VCI, PRI, WMI and PSI), the second set includes 10 subtests scores, and the last set includes 7 process scores. One common analysis is multivariate regression, which is essentially an ensemble of several multiple linear regression (Lambert et al., 1988). However, multivariate regression is not optimal due to the small sample size of this study (i.e., *n* = 45). For this reason, we employed RDA, which can effectively reduce the number of functionally independent parameters (Schmidli, 2013; Velu and Reinsel, 2013). Conceptually, RDA is similar to principal component regression (PCR), in which the criterion variable is regressed on a small number of principal components and reduces the number of estimated regression coefficients. However, PCR has a major limitation in that the retained principal components may not have sufficient explanatory power of the criterion variable (Jolliffe, 1982; Hadi and Ling, 1998). In contrast, RDA overcomes the limitation of PCR since the redundancy variates are constructed to have maximal explanatory power of the criterion variables (van den Wollenberg, 1977). Mathematically, the number of redundancy variates that can be constructed is up to the number of predictor variables, but typically it is sufficient to use a small number of redundancy variates to predict the criterion variables. The explanatory power of each redundancy variate is measured by an individual redundancy index, which is essentially the average R^2^ obtained by regressing each of the criterion variables on a single redundancy variate. Further, the obtained redundancy index is actually the maximal average R^2^ that one can possibly get. In short, relative to multivariate regression, RDA is a more parsimonious method, producing more stable parameter estimates (i.e., smaller standard error estimates), and thus appropriate for the analysis of small data. In this study, three sets of variables were created, with each set having multiple criterion variables and multiple predictor variables. Among these three sets, multiple predictor variables are all same across three sets while multiple criterion variables are different (e.g., set 1 includes 7 index scores; set 2 includes 10 subtest scores; set 3 includes process scores).

The IBM SPSS Statistics 27 program was used for most of the analyses other than redundancy analysis. Redundancy analyses were performed in SAS version 9.4 using the IML procedure.

## Results

### Sociodemographic and clinical characteristics

Sociodemographic characteristics of the BD-I and HC groups are presented in Table 1. There were significant demographic differences in age, years of education, average monthly income, and occupational status between the two groups. The BD-I group were significantly older, had fewer years of education and monthly income compared to the HC group. Regarding occupational status, in the BD-I group, the unemployed (33.3%) status was the highest, followed by students (17.8%), part-time positions (15.6%), and day laborers (15.6%). On the other hand, in the HC group, the student (32.6%) status was the highest, followed by full-time position (15.2%), and others (15.2%). There were no significant differences in gender and relationship status.

**Table 1:** Sociodemographic characteristics of the clinical sample and healthy controls.

	Bipolar disorder		Healthy controls		Group Comparison
	*n*	*Mean (SD)* or *n* (%)	*n*	*Mean (SD)* or *n* (%)	F or x^**2**^	*value of p*
Age	45	32.16 (8.85)	46	28.65 (5.91)	11.69	0.029
Gender	45		46		0.88	0.348
Male		22 (48.9%)		18 (39.1%)		
Female		23 (51.1%)		28 (60.9%)		
Years of education	45	13.13 (2.06)	46	15.80 (1.07)	23.68	<0.001
Average monthly income (USD)	35	3579.18 (1949.63)	45	4962.63 (2401.09)	2.33	0.007
Relationship Status	45		46		3.40	0.334
Single		33 (73.3%)		38 (82.6%)		
Married/Cohabiting		9 (20.0%)		8 (17.4%)		
Divorced/Separated		2 (4.4%)		0 (0.0%)		
Other		1 (2.2%)		0 (0.0%)		
Occupation	45		46		21.64	0.003
Unemployed		15 (33.3%)		9 (19.6%)		
Employed – full-time position		6 (13.3%)		7 (15.2%)		
Employed – part-time position		7 (15.6%)		2 (4.3%)		
Employed – day laborer		7 (15.6%)		0 (0.0%)		
Self-employed		0 (0.0%)		0 (0.0%)		
Homemaker		1 (2.2%)		5 (10.9%)		
Student		8 (17.8%)		15 (32.6%)		
Professional		0 (0.0%)		1 (2.2%)		
Others		1 (2.2%)		7 (15.2%)		

Group differences were analyzed using t-tests for continuous variables (i.e., age, years of education, average monthly income) and Chi-square test for nominal/ordinal variables (i.e., relationship status, occupation).

Additionally, Table 2 shows clinical characteristics of the BD group which were divided into ‘history’ and ‘current’ status. History section includes age of onset (*M* = 22.80, *SD* = 6.34), duration of illness (*M* = 9.36, *SD* = 8.83), type of the first episode (depressive type = 44.4%, manic type = 55.6%), past history of hospitalization (yes = 97.8%, no = 2.2%) and suicide attempts (yes = 37.8%, no = 62.2%). Current status section includes the severity of BD sample’s current symptoms, which was evaluated based on the MINI’s standard of module D (i.e., (hypo) manic episode) specifier (mild = 8.9%, moderate = 2.2%, severe without psychotic features = 57.8%, severe with psychotic features = 31.1%), current suicide risk based on the MINI module C (i.e., suicidality)‘s total points (none = 71.1%, mild = 4.4%, moderate = 8.9%, severe = 15.6%), and the scores on CGI-BP-depression (*M* = 4.76, *SD* = 1.58), CGI-BP-mania (*M* = 4.87, *SD* = 1.49), BRIAN (*M* = 45.76, *SD* = 11.47), QUIDS (*M* = 11.67, *SD* = 6.84), K-BAI (*M* = 13.00, *SD* = 14.57), K-MDQ (*M* = 8.64, *SD* = 4.01), BHS (*M* = 4.91, *SD* = 5.12), and BIS-T (*M* = 61.64, *SD* = 13.57) score. Lastly, the overall rate of current psychotropic medication is described. All of the BD-I participants were under psychotropic medication (*N* = 45, 100.0%) and the following are information for each psychotropics; Second-Generation Antipsychotics (SGA; *N* = 41, 91.1%), Mood Stabilizer (MS; *N* = 43, 95.6%), Benzodiazepine (BDZ; *N* = 34, 75.6%), and Antidepressant (AD; *N* = 0, 0.0%).

**Table 2:** Clinical characteristics of bipolar disorder (BD) samples.

	Bipolar Disorder (*n* = 45)
	*Mean (SD)* or *n* (%)
*Clinical variables - history*	
Age of Onset	22.80 (6.34)
Duration of Illness	9.36 (8.83)
Type of the First Episode	
Depressive type	20 (44.4%)
Manic type	25 (55.6%)
Age Received First Psychiatric Intervention	23.73 (6.51)
Past History of Hospitalization	
Yes	44 (97.8%)
No	1 (2.2%)
Age of First Hospitalization (*n* = 43)	24.72 (6.62)
Number of Past Hospitalization (*n* = 44)	4.25 (6.19)
Past Suicide Attempts	
Yes	17 (37.8%)
No	28 (62.2%)
Age of First Suicide Attempt (*n* = 17)	23.41 (9.56)
*Clinical variables - current*	
Severity	
Mild	4 (8.9%)
Moderate	1 (2.2%)
Severe without psychotic features	26 (57.8%)
Severe with psychotic features	14 (31.1%)
Current Suicide Risk	
None	32 (71.1%)
Mild	2 (4.4%)
Moderate	4 (8.9%)
Severe	7 (15.6%)
CGI-BP	
CGI-BP-depression	4.76 (1.58)
CGI-BP-mania	4.87 (1.49)
BRIAN	45.76 (11.47)
QIDS	11.67 (6.84)
BAI	13.00 (14.57)
MDQ	8.64 (4.01)
BHS	4.91 (5.12)
BIS-T score	61.64 (13.57)
Current Psychotropic Medication	45 (100.0%)
Second-Generation Antipsychotics (SGA)	41 (91.1%)
Mood Stabilizer (MS)	43 (95.6%)
Antidepressant (AD)	0 (0.0%)
Benzodiazepine (BDZ)	34 (75.6%)

CGI-BP, Clinical Global Impressions Scale-Bipolar Version; BRIAN, Biological Rhythms Interview of Assessment in Neuropsychiatry; QIDS, Quick Inventory of Depressive Symptomatology; BAI, Beck Anxiety Inventory; MDQ, Mood disorder questionnaire; BHS, Beck Hopelessness Scale; BIS, Barratt Impulsiveness Scale.

### Group differences in WAIS-IV performance

MANCOVA was conducted to identify whether the group differences exist on the WAIS-IV scores between the BD and HC group, even when controlling for the demographic covariates, age, and years of education. Results of MANCOVA showed that the dependent variables composed of WAIS-IV scores were significantly different between the two groups [*F*(24, 64) = 2.68, *p* < 0.001, Wilk’s Lambda = 0.50, η^2^*_p_* = 0.50]. Then, each dependent variable was analyzed using the post-hoc Bonferroni-corrected alpha level *p* < 0.001. The results showed a significant main effect of group for most of the WAIS-IV scores. Specifically, the main effect of the group was significant for composite scores including FSIQ [*F*(1, 87) = 20.83, *p* < 0.001, η^2^*_p_* = 0.19], GAI [*F*(1, 87) = 7.17, *p* < 0.05, η^2^*_p_* = 0.08], CPI [*F*(1, 87) = 31.11, *p* < 0.001, η^2^*_p_* = 0.26], PRI [*F*(1, 87) = 7.02, *p* < 0.05, η^2^*_p_* = 0.07], WMI [*F*(1, 87) = 13.82, *p* < 0.001, η^2^*_p_* = 0.14], and PSI [*F*(1, 87) = 27.29, *p* < 0.001, η^2^*_p_* = 0.24]. The VCI score did not show a significant difference between the two groups. Among the subtest scores, there was a significant main effect of group on VP [*F*(1, 87) = 8.54, *p* < 0.001, η^2^*_p_* = 0.09], DS [*F*(1, 87) = 8.66, *p* < 0.001, η^2^*_p_* = 0.09], AR [*F*(1, 87) = 11.04, *p* < 0.001, η^2^*_p_* = 0.11], SS [*F*(1, 87) = 19.78, *p* < 0.001, η^2^*_p_* = 0.19], and CD [*F*(1, 87) = 23.09, *p* < 0.001, η^2^*_p_* = 0.21] scores. BDN [*F*(1, 87) = 5.57, *p* < 0.05, η^2^*_p_* = 0.06], DSB [*F*(1, 87) = 10.40, *p* < 0.001, η^2^*_p_* = 0.11] and LDSB [*F*(1, 87) = 11.72, *p* < 0.001, η^2^*_p_* = 0.12] scores showed significantly lower performance in BD-I group among the process scores. All scores were significantly lower for the BD group than for the HC group. The results of the MANCOVA are reported in Table 3.

**Table 3:** Group differences in the WAIS-IV index, subtest, and process scores.

	Bipolar disorder (*n* = 45)		Healthy controls (*n* = 46)			
Test					*F*	*value of p*	η^2^_p_
	*Mean*	*SD*	*Mean*	*SD*					
*Composite scores*							
FSIQ	85.36	14.17	106.89	11.75	20.83	0.00	0.19
GAI	92.36	14.15	106.26	11.37	7.17	0.01	0.08
CPI	79.13	13.97	105.17	13.70	31.11	0.00	0.26
*Subtest scores*							
VCI	97.22	12.03	107.61	11.72	3.53	0.06	0.04
SI	9.60	2.55	11.00	2.52	3.06	0.08	0.03
VC	8.96	2.76	10.83	2.53	1.32	0.25	0.01
IN	9.60	2.48	11.85	2.55	3.13	0.08	0.03
PRI	90.18	16.01	104.35	11.54	7.02	0.01	0.07
BD	8.09	3.32	10.24	2.63	2.89	0.09	0.03
MR	8.89	2.86	10.91	2.05	3.23	0.08	0.04
VP	7.96	2.96	10.63	2.54	8.54	0.00	0.09
WMI	86.42	13.86	103.35	11.51	13.82	0.00	0.14
DS	7.78	2.71	10.59	2.23	8.66	0.00	0.09
AR	7.20	2.56	10.28	2.94	11.04	0.00	0.11
PSI	80.38	14.41	106.33	15.81	27.29	0.00	0.24
SS	6.09	2.70	10.89	3.54	19.78	0.00	0.19
CD	6.04	2.86	10.85	3.44	23.09	0.00	0.21
*Process scores*							
BDN	8.04	3.15	10.60	2.40	5.57	0.02	0.06
DSF	8.58	2.79	10.93	3.09	4.56	0.04	0.05
DSB	8.00	2.66	10.87	3.19	10.40	0.00	0.11
DSS	8.22	2.70	9.39	1.94	0.08	0.78	0.00
LDSF	6.91	1.16	7.85	1.23	3.70	0.06	0.04
LDSB	4.49	1.47	6.24	1.32	11.72	0.00	0.12
LDSS	4.89	1.34	5.63	1.10	0.02	0.88	0.00

FSIQ, Full Scale IQ; VCI, Verbal Comprehension Index; PRI, Perceptual Reasoning Index; WMI, Working Memory Index; PSI, Processing Speed Index; GAI, General Ability Index; CPI, Cognitive Proficiency Index; SI, Similarities; VC, Vocabulary; IN, Information; BD, Block Design; MR, Matrix Reasoning; VP, Visual Puzzles; DS, Digit Span; AR, Arithmetic; SS, Symbol Search; CD, Coding; BDN, Block Design No Time Bonus; DSF, Digit Span Forward; DSB, Digit Span Backward; DSS, Digit Span Sequencing; LDSF, Longest Digit Span Forward; LDSB, Longest Digit Span Backward; LDSS, Longest Digit Span Sequencing.

### Effects of clinical characteristics on WAIS-IV cognitive profile in BD group

**Criterion set 1**. In criterion set 1, seven redundancy variates and indices that have explanatory power on the criterion variables were created. Correspondingly, cumulative redundancy from the individual redundancy indices was computed. The first redundancy index of the first redundancy variate was 0.246, which means that 24.6% of the variance of criterion set 1 can be explained by the first redundancy variate (see table 4). The second redundancy index was 0.039, which means 3.9% of the variance of criterion set 1 can be explained by the second redundancy variate. Therefore, we only retained the first redundancy index for Set 1 considering the small proportion of variance of the remaining 6 redundancy variates. To interpret the first redundancy variate, redundancy loadings, which are the correlates between the original predictor variables and the retained redundancy variate, were utilized (see Table 5). Note that the signs of redundancy loadings are arbitrary, and it is the magnitude of redundancy loadings that matters. We can say that the predictor variables whose loadings share a same sign have the same direction on the redundancy variate, and it does not mean the positive or negative relationship like in zero-order correlation coefficient. The current study used 0.3 as a cut-off value, as it is a widely used criteria judged as ‘significant’ in the factor analysis context (Nunnally, 1978). Based on this cutoff value, symptom severity, CGI-mania, BRIAN, QIDS, BAI, and BHS showed a relatively larger association, in the same direction, with the first redundancy variate than other predictor variables such as psychotic features, age of onset, duration of illness, CGI-depression, MDQ, and BIS-T. Then, the cross-loadings, which are the correlations between the redundancy variates and the original criterion variables, are presented in Table 6. Besides PSI, the other six cross-loadings were all over 0.3 and the signs were all the same. Thus, the first redundancy variate was strongly and negatively associated with each criterion variable except PSI. As such, symptom severity, CGI-mania, QIDS, BAI, and BHS were more strongly associated with each criterion variable except PSI than other predictor variables.

**Table 4:** Individual and cumulative redundancy index.

Set 1		Set 2		Set 3	
Individual redundancy index	Cumulative redundancy	Individual redundancy index	Cumulative redundancy	Individual redundancy index	Cumulative redundancy
0.246	0.246	0.171	0.171	0.275	0.275
0.039	0.286	0.050	0.220	0.052	0.327
0.025	0.311	0.028	0.249	0.029	0.356
0.020	0.331	0.027	0.275	0.024	0.380
0.000	0.331	0.016	0.291	0.008	0.388
0.000	0.331	0.010	0.300	0.004	0.392
0.000	0.331	0.007	0.307	0.001	0.393
		0.002	0.309		
		0.001	0.310		
		0.000	0.310		

**Table 5:** Weight coefficients and redundancy loadings of the first redundancy variate.

	Set 1			Set 2			Set 3		
Predictor variables					
	Weight coefficients	Redundancy loadings	Weight coefficients	Redundancy loadings	Weight coefficients	Redundancy loadings
Severity	0.127	0.483	0.087	0.464	0.191	0.537
Psychotic features	0.158	0.287	0.154	0.264	0.293	0.487
Age of onset	0.035	−0.069	0.029	−0.057	0.054	−0.165
Duration of illness	0.481	0.169	0.526	0.215	0.641	0.401
CGI-depression	−0.029	−0.072	−0.021	−0.054	−0.163	−0.174
CGI-mania	0.720	0.455	0.783	0.493	0.502	0.318
BRIAN	−0.127	0.365	−0.157	0.295	0.328	0.273
QIDS	−0.074	0.347	−0.088	0.279	−0.230	0.159
BAI	0.267	0.498	0.212	0.431	0.034	0.357
MDQ	0.020	−0.152	−0.039	−0.226	−0.265	−0.293
BHS	0.677	0.478	0.674	0.442	0.255	0.226
BIS-T	0.346	0.296	0.390	0.240	0.412	0.288

CGI-depression, Clinical Global Impressions Scale-depression version; CGI-mania, Clinical Global Impressions Scale-manic version; BRIAN, Biological Rhythms Interview of Assessment in Neuropsychiatry; QIDS, Quick Inventory of Depressive Symptomatology; BAI, Beck Anxiety Inventory; MDQ, Mood disorder questionnaire; BHS, Beck Hopelessness Scale; BIS, Barratt Impulsiveness Scale.

**Table 6:** Cross-loadings of the first redundancy variate.

Set 1		Set 2		Set 3	
Criterion variables	Cross-loadings	Criterion variables	Cross-loadings	Criterion variables	Cross-loadings
FSIQ	−0.564	BD	−0.379	BDN	−0.564
GAI	−0.562	SI	−0.335	DSF	−0.562
CPI	−0.474	DS	−0.521	DSB	−0.474
VCI	−0.476	MR	−0.523	DSS	−0.476
PRI	−0.502	VC	−0.597	LDSF	−0.502
WMI	−0.564	AR	−0.501	LDSB	−0.564
PSI	−0.265	SS	−0.285	LDSS	−0.265
		VP	−0.386		
		IN	−0.219		
		CD	−0.142		

FSIQ, Full Scale IQ; VCI, Verbal Comprehension Index; PRI, Perceptual Reasoning Index; WMI, Working Memory Index; PSI, Processing Speed Index; GAI, General Ability Index; CPI, Cognitive Proficiency Index; SI, Similarities; VC, Vocabulary; IN, Information; BD, Block Design; MR, Matrix Reasoning; VP, Visual Puzzles; DS, Digit Span; AR, Arithmetic; SS, Symbol Search; CD, Coding; BDN, Block Design No Time Bonus; DSF, Digit Span Forward; DSB, Digit Span Backward; DSS, Digit Span Sequencing; LDSF, Longest Digit Span Forward; LDSB, Longest Digit Span Backward; LDSS, Longest Digit Span Sequencing.

**Criterion set 2**. In criterion set 2, ten redundancy variates and corresponding redundancy indices from the same predictor variables were created (see Table 4). The first redundancy variate explained 17.1% of the variance of criterion set 2. Since the remaining variates explained less than 5% of the variance, they were excluded from further analysis. Based on the redundancy loadings of the first redundancy variate, severity, CGI-mania, BAI, and BHS showed a higher value than 0.3 and demonstrated significant association with the redundancy variate. The cross-loadings of the redundancy variate showed that besides SS, IN, and CD among 10 subtests of the WAIS-IV, the other seven subtests presented higher values than 0.3 and the signs were all the same. Thus, the predictor variables including severity, CGI-mania, BAI, and BHS were more strongly associated with each seven criterion variable (i.e., BD, SI, DS, MR, VC, AR, and VP).

**Criterion set 3**. In criterion set 3, the first redundancy variate explained 27.5% of the variance of criterion set 3 (see Table 4). According to the redundancy loadings of the first variate, severity, psychotic features, duration of illness, CGI-mania, and BAI were significantly associated with the redundancy variate (see Table 5). The cross-loadings of the redundancy variate showed that the variate was associated with all criterion variables except LDSS, which showed a smaller value than 0.3 (see Table 6). Consequently, clinical features including severity, psychotic symptoms, duration of illness, CGI-mania, and BAI explain most of the performance of WAIS-IV process scores.

## Discussion

The present study sought to evaluate the cognitive profiles of the patients with BD-I, current or most recent episode manic, utilizing the full version of the WAIS-IV by comparing their profiles with the HC group. Although research indicates neurocognitive deterioration in bipolar disorder, their full cognitive profiles are rarely measured, which could identify the patients’ intact and impaired areas of function. Furthermore, the current study aimed to investigate which clinical characteristics— including current status and past clinical history—would explain the most of cognitive performance of BD-I patients. As such, it was hypothesized that (1) BD-I group would demonstrate significantly lower scores on the WAIS-IV profile, specifically on the scores related to working memory and processing speed, while abilities related to verbal comprehension and perceptual reasoning area are preserved, and (2) specific clinical characteristics would explain BD group’s cognitive function more than other clinical variables.

While most of the previous research evaluated the neurocognitive function of BD-I patients with different sets of testing batteries which hinders accurate comparison of study results (Douglas et al., 2018), the current study provides clear information of cognitive profiles of BD-I patients since the WAIS has been widely utilized in clinical settings. Although there are previous studies that utilized the previous version of WAIS (i.e., WAIS-III; Matsuo et al., 2021) or used only several selected subtests from the WAIS-IV for bipolar disorder group (Bo et al., 2019), to our knowledge, this is the first empirical evidence to provide the cognitive profiles of patients with BD-I, specifically whose current or most recent episode was manic. The results showed significant differences between BD-I and HC groups in the areas related to working memory and processing speed, which in turn negatively affected overall cognitive proficiency (i.e., CPI score) and full-scale IQ in the BD-I group. Specifically, the BD-I group demonstrated impairments in all subtests related to working memory (i.e., Digit Span, Arithmetic) and processing speed (i.e., Symbol Search, Coding). Although the BD-I group did not show any other significant impairments in verbal comprehension and perceptual reasoning domains, significant deterioration in Visual Puzzles (VP) subtest was observed, which is in the perceptual reasoning domain. For VP, more complex cognitive and mental manipulations are required such as mental set shifting *via* visualization, which utilizes abilities of visual working memory and sustained attention. Therefore, although the effect size of VP was smaller than other deteriorated areas, these results represent that the abilities measured through VP are significantly impaired in BD-I patients. Furthermore, other than VP, most of the effect size of these impairments when compared to HC group is characterized as large for both working memory and processing speed domain, based on the effect size values of the partial η^2^ (Cohen, 1973). Particularly, the processing speed domain was the most deteriorated area. These findings align with previous research that showed a significant decrease in processing speed, short-term memory, concentration, attention, and manipulation of information during manic episodes and even during euthymic status (Cardenas et al., 2016; Bora, 2018). These results also align with the previous studies that utilized the previous version of the WAIS, where BD-I patients demonstrated a significant decline in symbol coding subtest (Daban et al., 2012; Sparding et al., 2015).

In contrast, there was no significant difference between BD-I and HC groups regarding all verbal comprehension related subtests and most of the perceptual reasoning related subtests. These results imply that linguistic and logical reasoning ability, verbal comprehension and expression ability, and general knowledge acquired through past educational experiences were intact in BD-I group. Indeed, these findings suggest the BD-I groups’ preserved crystallized intelligence (Horn, 1965), which could reflect less impact from mood disorder symptoms or progress. However, given that cognitive efficiency is still significantly lower, which enables optimal use of mental resources for learning or problem solving (Hoffman, 2012), these results suggest that intact cognitive abilities including verbal comprehension and perceptual reasoning may not be fully utilized in BD-I patients in their daily life due to the deteriorated function in cognitive proficiency.

Regarding the second aim, our results suggested specific clinical features that can explain most of the cognitive performances of BD-I group. Redundancy coefficients demonstrated variance explained for cognitive performance in BD-I patients by clinical features incorporated with current clinical status (e.g., clinical severity, self-and clinician-reported measures) and past clinical history (e.g., age of onset, duration of illness), which were in the 17.1–27.5% range depending on the criterion sets of the WAIS-IV. Specifically, the process scores set were explained the most (i.e., 27.5% of the variance) by clinical features, followed by index scores set (i.e., 24.6% of the variance) and subtest scores set (i.e., 17.1% of the variance). Furthermore, although the magnitude of individual predictors’ effects accounted for cognitive performance varied across three groups (i.e., index, subtest, and process score group), three common predictors were identified as follows: overall symptom severity, manic symptom severity, and anxiety level. These results indicate that in BD-I patients, most of the cognitive domains related to verbal comprehension, perceptual reasoning, and working memory could be significantly impacted by symptom severity and anxiety. These findings align with previous studies investigating risk factors of cognitive deterioration in bipolar disorders, suggesting overall symptom severity as a crucial variable that negatively impacts cognitive performance (Zhu et al., 2019). The current results extend the previous research underscoring that, in addition to reductions in manic symptoms of BD-I patients, anxiety also requires more attention in intervention procedures as it was demonstrated to have a significant impact on overall cognitive performance. Even though bipolar disorder is regarded as one of the main mood disorders, patients with bipolar disorder often suffer from anxiety symptoms (Goldberg and Fawcett, 2012; Goes, 2015) and comorbidity between bipolar disorders and anxiety disorders is considerably high (Pavlova et al., 2015; Yapici Eser et al., 2018), which underlines the need for monitoring and addressing anxiety symptoms in patients with bipolar disorders. Indeed, recent studies on the impact of anxiety symptoms in bipolar disorder demonstrated unfavorable outcomes or a worse prognosis (Lorenzo-Luaces et al., 2018; Spoorthy et al., 2019; Kim et al., 2021). For instance, a significant association between anxiety symptoms and gray matter volume deficits in the left middle frontal lobe was presented, which implies neural evidence of the anxiety symptoms in BD (Song et al., 2020). Furthermore, there were predictors whose associations with cognitive domains are minimal, such as age of onset, depression severity, past mood disorder history, and impulsiveness. These findings are critical considering the confounding results on risk factors of cognitive deteriorations of bipolar disorder.

Although the results suggest potential risk factors that explain BD-I patients’ cognitive performance the most, the support for processing speed (cross-loading = −0.265) was weaker compared to those of other indices including verbal comprehension (−0.476), perceptual reasoning (−0.502), and working memory (−0.564). Considering that the most impaired cognitive area is processing speed among BD-I patients as shown in this study and previous research, these findings suggest that additional factors that were not included in this study may emerge as more significant risk factors that explain the deficit in processing speed area the most. Since little work has evaluated clinical features that directly explains processing speed deterioration, this represents an area for future research, as processing speed is closely associated with specific daily living activities and occupational function in patients with bipolar disorders (Anaya et al., 2016; Duarte et al., 2016; Solé et al., 2018).

When considering these results from the statistical perspective, the redundancy analysis (RA) provides strong support for the results. Traditionally, researchers conducted multiple regression analysis for each of the criterion variables when there were multiple predictors and criterion variables as this study. However, these analyses do not allow researchers to identify how the predictor variables are related to all the criterion variables at the same time, on top of increasing errors due to multiple analyses. RA overcomes this limitation since it provides the maximal explanatory power (i.e., redundancy index) on all criterion variables (van den Wollenberg, 1977). Therefore, RA is an appropriate method than any other method (e.g., multiple regression analysis, PCR, or multivariate regression analysis) for the analysis of multivariate data in terms of explanatory power in detecting the relationship between variable sets and parsimony.

### Limitations and strengths

The current study has some limitations. First, participants were recruited from a single medical institution and the sample size was small, which may have affected the representativeness of BD-I patients and HC group. Future research should aim to confirm the generalizability of the current results with a larger and more diverse population. Second, the data collection was conducted cross-sectionally regarding both cognitive function and clinical characteristics. Longitudinal research is required to investigate the ongoing interaction between cognitive deterioration and clinical features, and evaluate whether and how the specific risk factors such as manic symptoms severity, anxiety, or hopelessness continuously impact cognitive performance. Lastly, the present study could not conduct further analyses of the psychotropic effect on cognitive function, as the majority of the BD-I participants were under pharmacological treatment. The substantial differences in sample size between medicated versus non-medicated groups are likely to violate the assumption of equal variance, increase the type I error rate, decrease the statistical power, and thus, negatively affect the validity of the statistical analyses (Rusticus and Lovato, 2014). We could not also include psychotropic medication in RDA, in which the primary assumption is using only continuous variables, as the inclusion of binary variables is likely to violate the assumption of equal variance of random variables. Furthermore, while typical or first-generation antipsychotics are well-known for their negative impact on cognitive function, the current study’s participants were all under second-generation/atypical antipsychotics, which are known to have a significantly less negative impact or even positive impact on cognitive performances (Goldberg et al., 2007; Hill et al., 2010; Nielsen et al., 2015).

Despite these limitations, the present study also has several strengths. This study utilized the latest version of the WAIS to identify cognitive profiles of the BD-I group and compared it with HC group. This approach to the BD groups’ cognitive performance is novel in that there is a lack of previous studies that evaluated most of the cognitive domains, which includes crystallized and fluid intelligence that can be covered *via* WAIS-IV verbal comprehension and perceptual reasoning index. Second, methodologically, this study utilized redundancy analysis which has multiple advantages when there are both multiple predictor variables (e.g., clinical features) and criterion variables (e.g., WAIS-IV scores). The results from this multivariate analysis technique provided risk factors that explains the BD groups’ cognitive performance the most, while decreasing the error that can arise when multiple times of regression analyses are conducted. Third, clinical features of patients with BD-I were evaluated in a multifaceted way. Specifically, both clinicians and patients themselves assessed the severity of various symptoms (e.g., CGI scored by the clinician, MDQ scored by the patient) which allowed avoidance of biases and obtained balanced evaluation on the current status of patients. Furthermore, both subjective (e.g., symptom severity) and objective clinical characteristics (e.g., age of onset, duration of illness) were incorporated in the analysis. Lastly, Among BD group participants, only ‘current or most recent episode manic’ patients were included rather than having a mixed BD population, which is known to have heterogeneous cognitive performance and clinical features (Sole et al., 2012; Karanti et al., 2020).

## Conclusion

The results of the present study demonstrate significant BD-I patients’ cognitive deterioration in the cognitive proficiency area consisting of working memory and processing speed, while generalized ability including verbal comprehension and perceptual reasoning skills remain intact compared to those with the control group. Furthermore, the benefits of multivariate analysis for multiple predictors and criterion data allowed us to figure out the individual clinical features (i.e., manic symptom severity, anxiety) that best explain cognitive performance in BD-I patients. Indeed, these findings emphasize the importance of intervention not only for manic symptoms but also for anxiety reduction, which may have been overlooked in mood disorder treatments. Considering that WAIS is one of the most popular assessment tools especially in clinical settings, these results of BD-I patients’ cognitive profile would provide information for evaluating the patients’ cognitive decline status, and furthermore, for clinical decision making whether the patient would need additional assistance for impairments in cognitive abilities.

## Data availability statement

The datasets presented in this article are not publicly available due to confidentiality regulations. Requests to access the datasets should be directed to psyleewh@korea.kr.

## Ethics statement

The studies involving human participants were reviewed and approved by the Institutional Review Board of the National Center for Mental Health [NCMH IRB 116271-2019-03], Seoul, South Korea. Participants were provided with written informed consent to participate in this IRB-approved research project. The patients/participants provided their written informed consent to participate in this study.

## Author contributions

HK and WL jointly conceptualized the idea for research and designed the paper. HK analyzed the data, drafted the initial manuscript, and involved in the editing and revisions. DP and VR were responsible for resources allocation, funding acquisition, and provided intellectual input to the manuscript. JS was responsible for data analysis, statistical consultation, and contributed to initial manuscript drafting and editing. RY was responsible for data collection and management. WL contributed to manuscript editing, provided supervision, and was involved in editing and revisions. All authors contributed to the article and approved the submitted version.

## Funding

This research was supported by an intramural grant from the NCMH—No. 2019–01.

## Conflict of interest

The authors declare that the research was conducted in the absence of any commercial or financial relationships that could be construed as a potential conflict of interest.

## Publisher’s note

All claims expressed in this article are solely those of the authors and do not necessarily represent those of their affiliated organizations, or those of the publisher, the editors and the reviewers. Any product that may be evaluated in this article, or claim that may be made by its manufacturer, is not guaranteed or endorsed by the publisher.
